# 

**Published:** 1889-10-19

**Authors:** 


### NOTICE TO CORRESPONDENTS.

All MS., Letters, Books for Review, and other matters intended for the Editor, should be addressed The Editor, The Lodge, Porchester Square, London, W.

**Notice to Advertisers and Subscribers.**

All Advertisements, Orders for Copies of The Hospital, and Business Communications should be addressed to The Publisher, *not to the Editor*, The Hospital, 139-140, Salisbury-court, Fleet-street, E.C.

Vol. V., now ready, 4s., post free. Cases for binding, may be had of the Publisher, at 1s. 6d. each.

**To Residents, Secretaries, and Matrons.**

The Editor will be glad to have the Regulations and each Report as issued by Asylums, Hospitals, Convalescent and Nursing Institutions, as well as those of other Charities, and an early copy in duplicate of all Appeals and Festival Notices, etc., addressed to *The Editor, The Lodge, Porchester-square, London, W.* Any superintendent, secretary, matron, or other chief official who regularly sends such information may be registered, on application to the Publisher, to receive free of cost a cover for binding The Hospital in half-yearly volumes.

### Scraps and Gleanings.

Leprosy is prevalent in Zululand.

Cholera is spreading in Mesopotamia.

Coleraine is going to have a cottage hospital.

There is an epidemic of dengue fever at Smyrna.

On Hospital Saturday at Grantham £47 was collected.

A book on "The Liver," by Lionel Beale, is just published.

The body of Sir Tindal Robertson was cremated at Woking.

A meeting has been held at Buxton in aid of the Cyprus Society.

Dr. Jeremiah Wase, of Huntsfield, committed suicide last week.

Hospital Saturday in Colchester resulted in a collection of £130.

The Royal Commission on Vaccination have resumed their sittings.

A new hospital to accommodate 200 patients has just been opened at Vienna.

Dr. Spottiswood Cameron has been appointed medical officer of health for Leeds.

Queen Elizabeth of Roumania has been doing a six weeks' cure under Dr. Metzger.

Dr. and Mrs. Meers have left Sunderland to undertake work as medical missionaries.

Dr. Tatham has condemned a block of houses in the centre of Manchester as unhealthy.

Dr. Leidesdorf, the eminent specialist for mental diseases in Germany, died last week.

A fine hospital has just been completed at Medicine Hat, Canada. This is as it should be.

Birmingham Dental Hospital has had its waiting-room beautifully decorated by amateurs.

The St. John Ambulance Association have awarded 140 certificates to students at Birkenhead.

Mr. Wardle laid the foundation-stone of the Rumworth Fever Hospital on October 2nd.

At Withington Workhouse a patient lately died from choking, having greedily eaten some potatoes.

On October 3rd, an interesting conversazione was given at the Brompton Hospital by the medical staff.

Miss Caroline Schultze, house surgeon to a Paris hospital, has married her colleague, Dr. Bertillon.

The "Lion" training ship for boys is in quarantine at Devonport, owing to the prevalence of scarlet fever.

Dr. Edmond has added to his other generous gifts to the Aberdeen Convalescent Hospital, a cheque for £200.

North Riding Infirmary reports a half-yearly income of £1,760, and an expenditure of £1,582. This is satisfactory.

The son and daughter of Sir William Roberts, physician, are leaving for South Africa to undertake missionary work.

Sir George Porter delivered the opening address to the students of Meath Hospital, giving much good and diverse advice.

Mr. Hamilton Hoare, of Fleet-street, has given a donation of £50 to the Royal Hospital for Diseases of the Chest, City-road.

Messrs. Gill and Co. have just issued a card headed "Sanitary Hints for Householders," by Mr. G. M. Lawford, Assoc.Inst.C.E.

A committee has been elected at Middlesbrough to further the interests of the Friendly Societies' Convalescent Home scheme.

In Wilton Hospital, near Manchester, there are now over 100 fever patients, though the nominal accommodation is only for 65 beds.

The Queen's gift of £3 has been forwarded to Mrs. Green, a gardener's wife at Jersey, who recently gave birth to triplets, all of whom are still living.

King Humbert has sent a donation of 40,000f. towards the relief fund for the victims of the recent terrible storm in the Italian province of Cagliari.

Dr. Donald J. Macintosh, now of Belvidere Hospital, has been appointed first resident surgeon and superintendent of Victoria Infirmary, Glasgow.

It is reported that a serious epidemic of diphtheria is raging at Salford, and that there is an almost entire absence of hospital accommodation.

The Minnehaha Amateur Minstrels of Manchester have given £879 to charities (chiefly hospitals) as the result of their last season's entertainments.

Truro kept Hospital Sunday on Oct. 6th. The Rev. Canon Moore preached, and highly praised the Royal Cornwall Infirmary and the Truro Dispensary.

Dr. W. H. Dawson, of Malvern, has been presented with a clock and a purse of sovereigns. Dr. Dawson has been connected with the Rural Hospital for twenty years.

The measles epidemic at Rugeley still prevails with unabated vigour, and is not now confined to one class of the inhabitants, for children of both rich and poor are infected.

Professor Isidor Neumann, the specialist for diseases of the skin, who was summoned from Vienna to Portugal to give his opinion on the King's condition, has declared it to be very serious.

The Birmingham and Mid-Counties branch of the British Medical Association have invited the parent body to hold their 1890 meeting at Birmingham. It is understood that the invitation will be cordially entertained.

At the Sanitary Congress Mr. C. E. Capel read a paper on "The Extension of Public Analysis," in which he spoke of the adulteration of articles of domestic use, and complained of the neglect of the carrying out of the Food and Drugs Act. Other speakers followed, contending that the persons to be prosecuted were not, as was generally the case, the small shopkeepers, who were for the most part ignorant of the fact of adulteration, but the original adulterators.

